# Structural divergence in protein evolution of a photoreceptor family undetected by AlphaFold is observed by high sensitivity FT-IR spectroscopy

**DOI:** 10.64898/2026.08.04.742864

**Published:** 2026-08-06

**Authors:** Rosalie L. Dohmen, Gunnar Hoogerwerf, Aihua Xie, Wouter D. Hoff

## Abstract

A universal mechanism in molecular evolution is functional and structural divergence of members of a protein family. The ability of AlphaFold to predict atomic-resolution protein structures promises to accelerate insights into this process. We study the interplay of changes in sequence, structure, and function in photoactive yellow protein (PYP), a family of bacterial blue light photoreceptors. *Halorhodospira halophila* contains two PYP homologs that diverged to 60% sequence identity, differ 100-fold in the lifetime (τ _pB_ ) of their pB signaling intermediate, and display altered peak wavelengths (λ _max_ ) for color sensing. We resurrected ancestral PYPs and determined these properties along the resulting recapitulating evolutionary divergence. The resurrected ancestral PYP is functionally similar to PYP1, indicating divergence on the path to PYP2. AlphaFold predictions for PYP2 and these ancestral proteins revealed the absence of structural changes compared to the crystal structure of PYP1. To experimentally validate these predictions, we optimized second-derivative Fourier transform infrared (FTIR) spectroscopy. The FTIR spectra of PYP1 and 2 and their resurrected ancestral proteins demonstrated clear differences in their secondary structure. These results demonstrate an important limitation of AlphaFold and show how ancestral sequence reconstruction combined with spectroscopic approaches yields insights into divergence in a protein family.

## Full Text Availability

The license terms selected by the author(s) for this preprint version do not permit archiving in PMC. The full text is available from the preprint server.

